# All-Optical Planar Polymer Waveguide-Based Biosensor Chip Designed for Smartphone-Assisted Detection of Vitamin D

**DOI:** 10.3390/s20236771

**Published:** 2020-11-27

**Authors:** Johanna-Gabriela Walter, Lourdes S. M. Alwis, Bernhard Roth, Kort Bremer

**Affiliations:** 1 Institute of Technical Chemistry, Leibniz University of Hannover, 30167 Hannover, Germany; walter@iftc.uni-hannover.de; 2 School of Engineering and the Built Environment, Edinburgh Napier University, Edinburgh EH10 5DT, UK; L.Alwis@napier.ac.uk; 3 Cluster of Excellence PhoenixD, (Photonics, Optics, and Engineering—Innovation Across Disciplines), 30167 Hannover, Germany; bernhard.roth@hot.uni-hannover.de; 4 Hannover Centre for Optical Technologies, Leibniz University of Hannover, 30167 Hannover, Germany

**Keywords:** surface plasmon resonance, biosensor, aptamers, vitamin D, optical waveguide, integrated optics

## Abstract

An all-optical plasmonic sensor platform designed for smartphones based on planar-optical waveguide structures integrated in a polymer chip is reported for the first time. To demonstrate the applicability of the sensor system for biosensing purposes, the detection of 25-hydroxyvitamin D (25OHD) in human serum samples using an AuNP-enhanced aptamer-based assay was demonstrated. With the aid of the developed assay sensitivity of 0.752 pixel/nM was achieved for 25OHD concentrations ranging from 0–100 nM. The waveguide structure of the sensor enables miniaturisation and parallelisation, thus, demonstrates the potential for simultaneous detection of various analytes including biomarkers. The entire optical arrangement can be integrated into a single polymer chip which allows for large scale and cost-efficient sensor fabrication. The broad utilization and access of smartphone electronics make the proposed design most attractive for its wider use in lab-on-chip applications.

## 1. Introduction

With the recent emergence of the Internet of Things (IoT), there is an increasing demand on unleashing the full potential of fibre optic sensor technology towards “mobile labs” or the so-called “lab-on-chip” applications. The concept of IoT depends solely on the inter-connectivity of devices within a cluster and this requires not only a communication platform that can handle vast amounts of data but also sensing/information-gathering platforms that are efficient and portable. In line with this paradigm shift, a newly emerging concept is the design of portable hand-held fibre optic sensors that can directly feed information onto smartphones [1]. Even more interesting is the possibility, via IoT, of having bio-sensors that can directly monitor specific disease-related parameters of an individual and the information is then sent to relevant authorities or the pharmacy, who can issue the appropriate prescription which will subsequently arrive at the patient’s doorstep. For such achievement, the first step would be to verify the possibility of sensors being connected to a smartphone and are suitable to be used by the end-user. In light of the fact that in 2019 approximately 3.2 billion people used a smartphone [2], i.e., representing over 40% of the world population, it can be expected that the demand for lab-on-chip sensors for smartphones is on a steady rise.

Since their release at the beginning of the 21st century, smartphones have evolved to their present state with excellent built-in technology, i.e., multi-core processors, open-source operating systems, multi-point touch screen recognition, digital cameras with high image resolution and extremely fast shutter speed, all of which makes them highly versatile and attractive for autonomous end-user biosensing applications. Notably, these potential applications are not restricted to healthcare diagnostics alone but are also attractive for food production and environmental monitoring sectors. Smartphones also allow the development of portable and point-of-care test capability outside of laboratory settings, thus the important possibility of offsite diagnosis. What is even more remarkable is the novel concept of utilizing integrated photonics within smartphones to design optical-based sensor platforms. For instance, by taking advantage of the integrated camera and flashlight LED, smartphones have been transformed to mobile spectral endoscopes [3] as well as fluorescent [4] and mobile holographic microscopes [5], among others, which render them suitable for mass-market products such as disposable lab-on-a-chip devices.

Amongst the many available sensing techniques for biosensor applications [6,7,8], Surface Plasmon Resonance (SPR) has established itself to be the gold standard in label-free biomarker detection [9] and holds potential to be further improved by the use of novel micro-and nanostructured materials [10,11]. Due to their surface wave properties, surface plasmons have a high sensitivity to changes in the dielectric function of the medium of propagation and thus possess the unique property of allowing the monitoring of biomarker binding in real-time. Furthermore, it was also demonstrated that SPR based sensors are capable of detecting biomarkers in the femtomolar (fM) range using gold nanoparticle (AuNP)-assisted sandwich assay approaches. For instance, Kim et al. [12] demonstrated the detection of IgE with a sensitivity down to 1 fM. The main issue that had limited the highly sensitive SPR technology to laboratory environments thus far is its requirements of bulky and expensive equipment. However, with the uptake of optical fibre-based sensing schemes, the classic SPR setup had seen a dramatic change where it can now be realised on the surface of optical fibres or optical waveguides [13], which allow, in comparison to traditional SPR equipment, mobile applications and have a higher potential of miniaturization. This has drastically changed the landscape of SPR-based biosensors within the past decade.

Due to their advantages, SPR based sensor platforms have also been reported for smartphones [14]. For instance, Guner et al. [15] reported a grating-based SPR sensor platform for smartphones with a bulk refractive index sensitivity of 4.12 × 10^−5^ RIU to monitor IgG with nanomolar (nM) sensitivity. In the interest of extending the application of SPR-based and smartphone-assisted biosensing, all-optical based SPR sensor platforms for smartphones would be highly beneficial due to their cost-effective production and robustness. In this case, no additional photonic components, i.e., no additional light sources and lenses etc., are required for the smartphone-based interrogation of the SPR sensor. The first embodiments of this type of SPR sensor platforms were developed by Bremer et al. [1] and Liu et al. [16] in 2015, where both systems were based on fibre optic SPR sensors.

Previously, the authors have demonstrated the use of a simple white light LED and a spectrophotometer for the interrogation of an SPR-based sensor for biomarker detection [17]. The design proposed in this study aims to progress a step further through the utilization of a smartphone to provide the appropriate interrogation photonics in tandem with a microfluidic chip equipped with a two-channel SPR sensing scheme for biomarker detection. This novel concept was realised by the design and implementation of a small and portable “sensor housing” unit that can be directly applied to a smartphone via which the target biomarker detection is achieved through the use of the bio-sensing design contained within the sensor housing and the electronics available from the smartphone. The proposed design not only reduces the source/detector electronics cost of the overall system but provides additional advantages such as a wider application of SPR outside the laboratory, portability, mass-production capability, multiple biomarker detection possibility and the potential for connection to IoT via the smartphone.

In light of the discussion above, this communication presents for the first time an all-optical based SPR sensor platform (SmartSens) that utilizes a planar-optical waveguide structure integrated into a polymer chip instead of discrete fibre optic components. The advantage of the planar-optical waveguide structure is that the whole optical arrangement of the smartphone-based SPR sensor system can be integrated into a single polymer sensor chip and thus the whole sensor system can be shrunk to only a few square centimetres footprint. Moreover, the polymer chip nature of the proposed sensor system allows large scale and thus cost-efficient sensor fabrication. The functionality of the sensor system was evaluated using an aptamer-based competitive assay for 25-hydroxyvitamin D (25OHD) as a model analyte. Due to the waveguide structure of the sensor that allows a high degree of miniaturization and parallelization, the sensor holds the potential for multiplexing applications for the simultaneous detection of various biomarkers. Thus, the multiplexing capability, in combination with easy read-out that can be accomplished with a conventional smartphone, could qualify the sensor system for home testing applications, e.g., monitoring of chronic diseases, as well as for differential diagnostics in low resource settings.

## 2. Sensor Design

**Figure 1:**
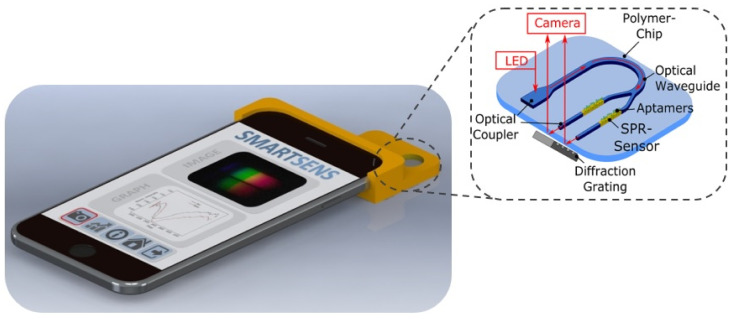
Conceptual design of the all-optical planar polymer-based biochip sensor platform for smartphones (SmartSens). As shown in the inset, a polymer chip containing planar-optical waveguides and Surface Plasmon Resonance (SPR) sensors can be interrogated using the flashlight LED and camera of a standard smartphone.

The proposed sensor concept is illustrated in Figure 1. The planar-optical waveguide-based SPR sensor chip is embedded in an appropriate sensor housing, which arranges the sensor system relative to the flashlight LED and camera at the back of the smartphone. When a photo is taken using the smartphone, light from the smartphone flashlight LED is coupled into the planar-optical waveguide structure of the SPR sensor chip and is guided further to a gold-coated SPR sensor region. At the SPR sensor region, the light interacts with the environment, i.e., with the specific analyte sample, resulting in the modification of light propagation, which is then guided further via a diffraction grating to the smartphone camera. As can be seen from the inset of Figure 1, the diffraction grating is an additional component which is placed in between the smartphone camera and the SPR sensor chip. The resulting photo of the diffracted light is then used to determine the SPR signal and thus to monitor the binding of the biomarker. It should be noted that the sensor chip can contain two or more SPR sensor elements and, therefore, multiplexed detection of two biomarkers could be performed.

To demonstrate the capability of the sensor system for biosensing applications, we demonstrate the detection of 25-hydroxyvitamin D (25OHD) as a model analyte from diluted serum samples. Since 25OHD is a relatively small molecule and thus only induces a relatively small refractive index change above the SPR sensor surface, a competitive aptamer-based assay recently developed by our team [18], was applied. In this assay, a DNA aptamer [19] directed against 25OHD is used in combination with an oligonucleotide that is complementary to a section of the aptamer which is involved in binding of the aptamer target. In the absence of the target, the oligonucleotide forms a duplex with the aptamer, while in the presence of 25OHD, the aptamer binds to its target instead. Through this process, the binding of the small molecule 25OHD, which is hard to detect by SPR in general, is translated into the dissociation of the larger oligonucleotide [18]. To further increase the 25OHD-induced changes of the refractive index above the sensor surface, gold nanoparticles (AuNPs) were used for signal enhancement, as described recently for aptamer-based detection of CRP [17].

## 3. Material and Methods

### 3.1. Fabrication of Planar-Optical Biosensor Chip

**Figure 2:**
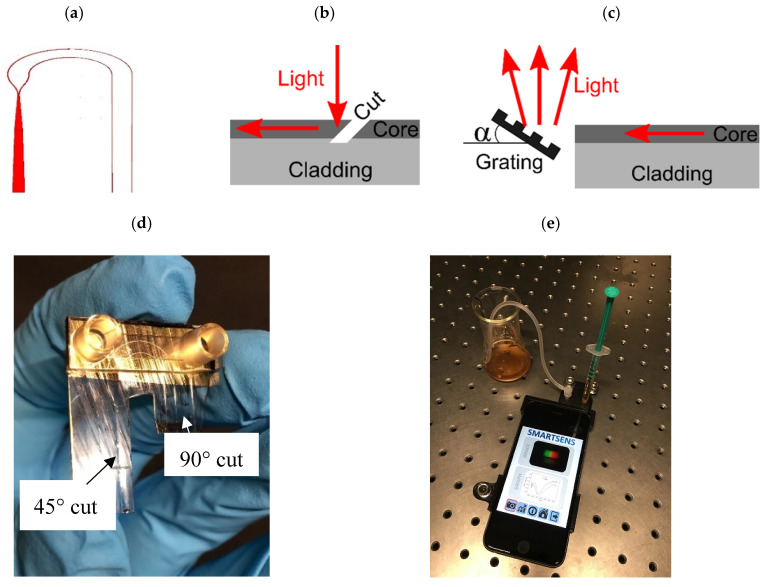
Schematic of the designed sensor system (**a**) as well as the applied light coupling structures to couple light into (**b**) and out of (**c**) the planar-optical waveguide sensor. For the light-in coupling a 45° cut and total internal reflection was applied, whereas for the light-out coupling a 90° cut and the diffraction grating in reflection mode was used. After assembling the coupling structures and the microfluidic (**d**), the sensor chip was embedded into a 3D printed sensor housing (**e**).

The planar-optical polymer sensor chip was designed in RSoft and all waveguide components have been modelled and optimised using the BreamProp toolbox for the use with an Apple iPhone 6s. The BreamProp toolbox is based on the Beam Propagation Method (BPM). A schematic of the designed and applied planar-optical waveguide structure is illustrated in Figure 2a. As illustrated in Figure 2a, the planar-optical waveguide structure starts (left side and aligned to the flashlight LED at the back of the smartphone) with a relatively broad waveguide segment (with a width of 1200 µm). This segment is applied to optimize the light coupling between the sensor chip and smartphone flashlight LED. Subsequently, the waveguide is tapered to reduce the waveguide width to 200 µm followed by a waveguide splitter to obtain two separate optical waveguides and thus to form two separate SPR sensors. After the splitter, the waveguide width of each channel was 100 µm and each waveguide was further tapered after the splitter element to obtain the final waveguide width of 50 µm. This particular waveguide width was chosen so that the resulting SPR sensor is comparable to our previous investigation of planar-optical SPR sensors [17]. Furthermore, a waveguide bend with an average radius of 5 mm was applied to align and guide the light from the flashlight LED to the camera of the smartphone. Based on this design concept, the planar-optical sensor chip was fabricated via the hot embossing manufacturing technique developed by Rezem et al. [20,21], which relies on hot embossing the optical waveguide structure with a depth of 25 µm into the polymer foils. For this, first, a silicon wafer stamp containing a negative copy of the desired structures is used to imprint the waveguide cladding structure into a 375 µm thick PMMA (refractive index of 1.49) sheet (Plexiglas XT 99524). Following the hot embossing, the doctor blading method was used to fill the cladding structure with optical epoxy material with a higher refractive index compared to PMMA and thus to form the optical waveguide. For the fabrication of the planar-optical sensor chip, the UV curable epoxy NOA 63 with a refractive index of 1.56 was applied. After the doctor blading, the epoxy material was cured using the UV light source (UV Transilluminator MUV21, Major Science, Saratoga, NY, USA).

Following this step, the part of the surface of the planar-optical waveguide structure containing the region of the two bent waveguides was coated with a thin titanium layer (2 nm) topped by a thin gold layer (40 nm layer thickness) to realize the SPR sensors. This part of the planar-optical waveguide structure was chosen since it simplifies the assembly with a microfluidic device. The thin titanium layer of the gold coating was applied to enhance the bonding between the polymer and the gold layer. Moreover, a microfluidic chip with one microfluidic channel was glued on top of the gold surface. In this instance, the microfluidic chip Ibidi sticky-Slide VI 0.4 was used and cut into individual channels. The channels used had a length of 17 mm, a width of 3.8 mm and a height of 400 µm. The fluid inlet and outlet of the microfluidic chip were standardised female Luer adapters. After the microfluidic chip was assembled, the planar-optical sensor system was cut into a geometry that fits the designed sensor housing for the Apple iPhone 6s (see e.g., Figure 2d).

### 3.2. Smartphone-Based Experimental Set-Up

#### 3.2.1. Optical Set-Up

The planar-optical biosensor chip was housed in a polymer containment to fix the polymer sensor to the smartphone (Apple iPhone 6s) as well as to conduct the experiments, i.e., see Figure 2e. The housing of the sensor system was 3D-printed using the Makerbot Replicator 2. It consists of two parts: The first part is required to align the sensor system relative to the smartphone flashlight LED and camera, which are placed at the bottom. The second part (placed on the top) was printed to protect the sensor from interrogation by environmental light.

The light coupling from the smartphone flashlight LED to the planar-optical sensor system and from the sensor system back to the smartphone camera was realised by the coupling structures shown in Figure 2b,c. For the light coupling into the sensor system, a 45° cut was applied, which was realised using a common razor blade. Due to the resulting air gap, the light is totally reflected at the polymer/air interface and thus coupled into the sensor system. The particular angle of 45° was chosen since it allows perpendicular light coupling between the smartphone flashlight LED and the planar-optical sensor system. For the light coupling from the sensor system back to the smartphone camera, the waveguides of the sensor system were cut perpendicular using a razor blade which was heated up to a temperature of 65 °C. Moreover, the light from the waveguide was guided onto a diffraction grating in reflection mode which then diffracts the light into the smartphone camera and thus allows the determination of the sensor spectrum. The diffraction grating was fabricated by replicating an 1800 lines/mm diffraction grating from Thorlabs (GH13-18V, Thorlabs, Newton, NJ, USA) using the hot embossing technique described above and sputtering of the resulting structure with a thin silver film (100 nm). The angle α in Figure 2c was 7° and achieved using 3D printing, i.e., the printing of the position for the grating was already incorporated in the 3D printing of the sensor housing. The applied diffraction grating period and angle were chosen to capture the whole light spectrum of the smartphone flashlight LED despite the limited aperture of the smartphone camera. For liquid handling, shrinks (B.Braun Injekt-F 1 mL), as well as a tube (Ibidi elbow male Luer adapter with silicone tubing), were connected to the Luer-adapters of the microfluidic chip of the sensor system. The liquids, to modify the gold surface of the SPR sensor, were applied using shrinks (B.Braun Injekt-F 1 mL) and the silicone tubing connected to the other end of the fluidic chip was used to guide the “waste” liquid away. An assembled sensor chip as well as the final experimental setup are depicted in Figure 2d,e.

#### 3.2.2. Data Analysis

To obtain the SPR response from the sensor chip, photo images were taken from the sensor system, i.e., the light coming from the final stage of the sensor chip through the diffraction grating, using the standard camera app from the iPhone while the flashlight LED acts as the light source. The camera was set to the infinity focus-mode and the integration time was set so that the obtained optical signal was not overexposed, i.e., that all colours are within the dynamic range of the detector. Following these settings, the photo images were grey-scaled to determine the intensity values per pixel and then the intensity distribution per column were calculated using Matlab. The intensity distribution per column represents the sensor spectrum. To reduce the signal noise, a moving average filter was applied (N = 300). Moreover, the obtained sensor spectrum was normalised to the light spectrum of the smartphone flashlight LED, which was taken at the beginning of every measurement before the sensor surface was incubated with aqueous solutions (i.e., the refractive index above the sensor surface would then be air, n = 1 where no SPR effect of the designed planar-optical sensor chip was expected or observed [17]). Following this step, the shift of the SPR wavelength was calculated using the “centre of mass” method [22] instead of only tracing the minimum SPR position. The “centre of mass” method was particularly chosen as it calculates a mean value, thus the measurement would be more robust, reliable and deems most suitable for the proposed sensing scheme.

### 3.3. Surface Functionalization to Detect Vitamin D

Surface modification was performed as described in [17]. In brief, the gold surface was coated with streptavidin by overnight incubation in a solution of 2.5 mg/mL streptavidin (Roth GmbH, Germany) in phosphate-buffered saline, pH 7.4 (PBS). Following thorough washing steps, the sensor was dried with compressed nitrogen and stored at 4 °C in a dry environment prior to use.

The single-stranded DNA-aptamer VDBA14: 5′-AGC-AGC-ACA-GAG-GTC-ATG-GGG-GGT-GTG-ACT-TTG-GTG-TGC-CTA-TGC-GTG-CTA-CGG-AA-3′ selected against 25OHD by Lee, Nguyen and Gu [20] was used with a 5′termial biotin modification (IDT) for functionalization of the streptavidin-coated sensor surface. Therefore, the biotinylated VDBA14 was diluted to 100 nM in binding buffer (BB) composed of 100 mM NaCl, 20 mM Tris-HCl, 2 mM MgCl_2_, 5 mM KCl, 1 mM CaCl_2_, pH 7.6 and incubated on the streptavidin-modified sensor surface for 1 h at room temperature. Afterwards, unbound aptamer was removed by rinsing the sensor with BB.

### 3.4. Production and Modification of Gold Nanoparticles

The AuNPs used for signal enhancement were prepared by the kinetically controlled seeded growth synthesis as described by Bastús et al. [23] with ten consecutive rounds of additions of HAuCl_4_ and sodium citrate. The size of the produced AuNPs was determined to be 17 nm via transmission electron microscopy (TEM) using a field-emission instrument of the type JEOL JEM-2100F-UHR. The AuNPs were modified with streptavidin as described before [17], by overnight incubation with 50 µg/mL streptavidin in 10 mM sodium phosphate pH 7.4.

### 3.5. Detection of Vitamin D

The aptamer-modified sensor was exposed to different concentrations of 25-hydroxyvitamin D. To obtain defined concentrations, commercially available 25-hydroxyvitamin D (Enzo Life Sciences, Farmingdale, New York, NY, USA) was diluted in 10% 25-hydroxyvitamin D-depleted human serum (VD-DDC Mass Spect Gold, Golden West Diagnostics, Temecula, CA, USA). Concentrations of 0 nM, 25 nM, 50 nM, and 100 nM 25-hydroxyvitamin D were prepared in BB supplemented with 10% Ethanol, 500 mM NaCl, and 10% 25-hydroxyvitamin D-depleted human serum. All solutions additionally contained 10 nM complementary oligonucleotide (sequence: AAA-GTC-ACA-C, [18]) with a 5′terminal biotin modification. The solutions were incubated on the sensor for 30 min to allow binding of 25-hydroxyvitamin D to the aptamer as well as hybridization of the oligonucleotide to those aptamer molecules that have not bound 25-hydroxyvitamin D under the given conditions. Subsequently, the sensor was rinsed with BB once, followed by an additional rinsing step with BB supplemented with 500 mM NaCl and a final rinse with ddH_2_O. Prior to the AuNP-assisted signal enhancement step, the streptavidin modified AuNPs were diluted in 25% BB to an absorption of 1 at the localised SPR maximum. The AuNPs were incubated on the sensor surface for 10 min before unbound AuNPs were removed by two rinses with ddH_2_O.

## 4. Results

### 4.1. Obtained Sensor Signals

**Figure 3:**
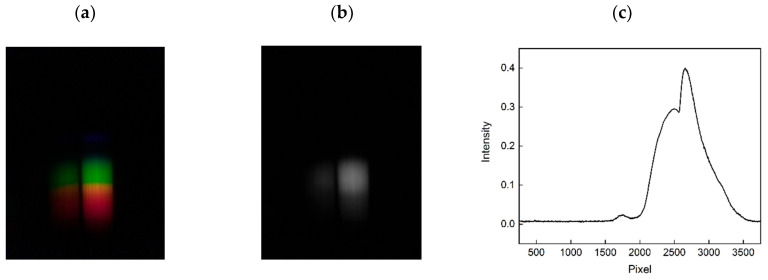
Detected spectra of the two SPR sensors using photo images from the smartphone (**a**) and subsequent grey-scaling (**b**). The grey-scaled image was then used to obtain the intensity values per column and thus to obtain the sensor spectra (**c**).

An example of an obtained photo image of the sensor system as well as the subsequent grey-scaling and determination of the sensor spectrum by calculating the average intensity values over neighbouring columns is illustrated in Figure 3. The measured sensor spectra are shown for the two adjacent optical waveguides of the polymer sensor chip in Figure 3a,b after the subsequent grey-scaling. As illustrated, the measured intensity spectrum of the first (left) waveguide is lower than that of the second (right) waveguide (see Figure 2a). The lower intensity of the first waveguide is due to the smaller waveguide bend. Therefore, the second waveguide was applied for the investigation of 25OHD detection. Moreover, after the grey scaling, the intensity distribution per column was calculated to obtain the sensor spectrum (Figure 3c). While the second waveguide was not used in the present study, it should be noted that after further optimization of the sensor design, several waveguides can be implemented in one sensor system which paves way for multiplexing applications in the future.

### 4.2. Determining the Sensitivity of the SPR Biosensor Chip to RI Changes

**Figure 4:**
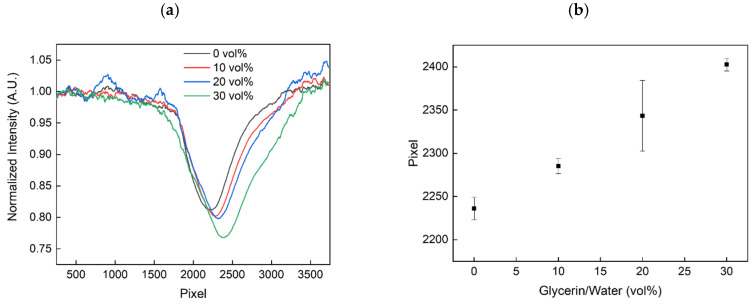
Measured sensor spectrum (**a**) and the resulting shift of the SPR (**b**) for increasing glycerin volume concentrations and thus for increasing refractive indices.

The performance of the SPR-based biosensor chip was first evaluated by measuring the response to different refractive index solutions, which were obtained using glycerin/water mixtures with varying glycerin volume concentrations (vol%). In total, four different glycerin/water solutions have been realised (0 vol%, 10 vol%, 20 vol% and 30 vol%). The measured sensor spectra, as well as the shift of the SPR due to different refractive index solutions, are illustrated in Figure 4a,b.

As illustrated in Figure 4, the SPR spectra could be measured and thus the response for increasing glycerin solutions, i.e., spectral shift, was obtained. The shift towards higher pixel numbers corresponds to a shift of the SPR towards higher wavelengths due to increasing refractive indices of the environment. These results agree well with our previously reported measurements from an SPR biosensor based on a polymer multi-mode optical waveguide [17], which indicated an SPR at 560 nm (n = 1.33) and a redshift of the SPR due to increasing refractive index solutions. Moreover, since no polarization filters were applied, only part of the guided light within the optical waveguide structure can couple to the surface plasmons. With the current configuration, a sensitivity of 5.67 pixels/vol% or 206.63 pixels/RIU (refractive index units) was achieved with a standard deviation of 12.79 pixels (0 vol%), 8.98 pixels (10 vol%), 40.84 pixels (20 vol%), and 7.34 pixels (40 vol%) and thus a Limit of Detection (LOD) of 0.19 RIU.

### 4.3. Evaluating the Detection of Vitamin D in Serum Samples

**Figure 5:**
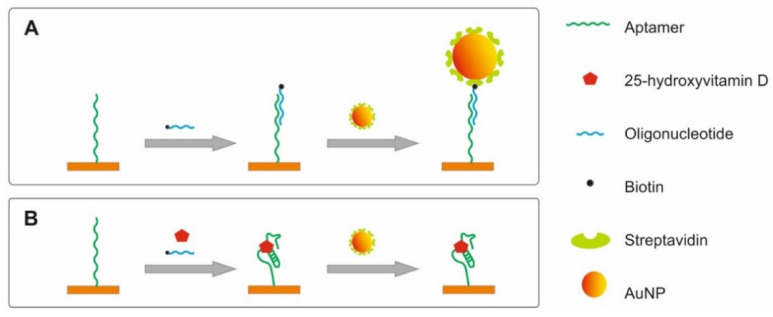
Schematic of the sensing strategy; Aptamers directed against 25-hydroxyvitamin D are immobilised on the sensor surface. In the absence of vitamin D, the aptamers hybridize with the complementary oligonucleotide. As the complementary oligonucleotide is biotinylated, it can bind streptavidin-modified AuNPs for signal enhancement (**A**). Aptamers that are bound to 25-hydroxyvitamin D do not facilitate hybridization of complementary oligonucleotide and binding of AuNPs (**B**).

The detection of 25-hydroxyvitamin D (25OHD) in spiked human serum samples using the developed planar-optical biosensor chip and the AuNP-enhanced competitive aptamer-based assay was evaluated. Here an oligonucleotide complementary to the aptamer and 25OHD competitively binds to the aptamer which is immobilised on the sensor surface. In absence of 25OHD, the oligonucleotide hybridizes with the aptamer, while in the presence of 25OHD, the target binds to the aptamer thereby preventing the hybridization of the oligonucleotide. Since the oligonucleotide is modified with biotin, it allows binding of streptavidin-modified AuNPs. In this assay format, high concentrations of 25OHD results in reduced binding of AuNPs to the sensor surface, while for low concentrations of 25OHD the AuNP density on the sensor surface increases. (The sensing principle is illustrated in Figure 5).

**Figure 6:**
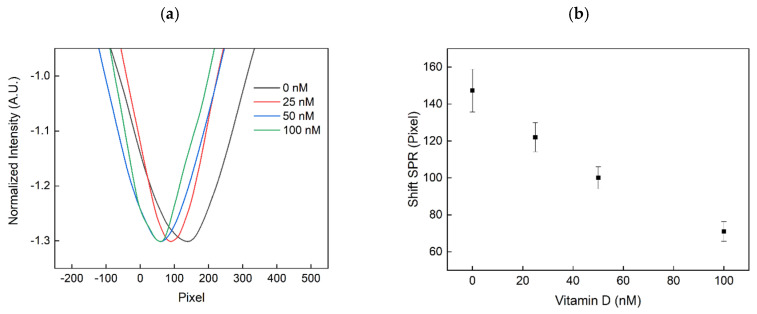
Measured sensor spectrum (**a**) and the resulting shift of the SPR signal obtained after calculating the position of the “centre of mass” of SPR sensor spectra for increasing Vitamin D concentrations (**b**).

The developed sensor and assay were investigated by measuring sensor response after incubation with different concentrations of 25OHD in 10% human serum. The SPR was measured before and after the sensing of 25OHD using water (n = 1.33) as a reference buffer. The obtained shift of the SPR is illustrated in Figure 6. Since a new sensor was applied for every vitamin D concentration (0 nM, 25 nM, 50 nM and 100 nM), the measured SPR spectrum was standardised to the reference measurement, i.e., the difference between the determined positions of “centre of mass” of SPR sensor spectra before and after immobilization was calculated for each sensor. Moreover, three measurements were performed for every vitamin D concentration. As depicted in Figure 6b, a sensitivity of –0.752 pixel/nM was obtained with a standard deviation of 8.05 pixel. The decrease in the SPR wavelength can be explained by the decrease of AuNPs that are immobilised on the gold surface of the SPR sensor, due to the increase in binding of the vitamin D molecule to the aptamers and thus decreased hybridization of the oligonucleotide (Figure 4). The sensor was capable of detecting 25 nM 25OHD in solution. As the desirable range of 25OHD in serum ranges from 50–100 nM [24], the sensing was successfully demonstrated for physiologically relevant concentrations.

Currently, 25OHD is conventionally detected in laboratories via various techniques such as liquid chromatography coupled with tandem mass spectrometry (HPLC-MS/MS), high-performance liquid chromatography with UV detection (HPLC-UV), or enzyme-linked immunosorbent assay (EIA), which share a limit of detection (LOD) of approximately 5 nM (approximately 2 ng/mL 25OHD) [25]. While the developed SPR sensor does not require a laboratory environment with sophisticated analytical devices, the SPR sensor can detect 25OHD in a concentration in the same range as for the above-mentioned state-of-the-art methods, thus highlighting the achievement of the novel approach presented herein. This indicates that the SPR sensor platform holds the potential to compete with conventional sensing techniques and to complement them in mobile applications.

## 5. Summary

An all-optical SPR sensor platform designed for smartphones based on planar-optical waveguide structures integrated into a single polymer chip is reported for the first time. As (i) all electronic/optical interfaces such as the light source/detector as well as signal processing and the power supply are provided by the smartphone (the proposed sensor chip is electrically passive) and (ii) the planar-optical polymer waveguide structure is optimised for large-scale sensor fabrication, the proposed sensor system is ideally suited for low-cost disposable point-of-care applications. The sensitivity of the proposed sensor system to different refractive index solutions was evaluated using different glycerin/water solutions. With the current setup, a sensitivity of 5.67 pixels/vol% or 206.63 pixels/RIU was achieved, respectively, with a LOD of 0.19 RIU. Moreover, to demonstrate the applicability of the sensor system for biosensing purposes, the detection of 25OHD in human serum was demonstrated using an AuNP-enhanced and aptamers-based competitive assay. With the aid of the developed assay, a sensitivity of −0.752 pixel/nM was achieved for 25OHD concentrations from 0–100 nM. Physiologically relevant concentrations of 25OHD could be detected that are in the same range as LOD of state-of-the-art methods.

The reported sensor chip design contains two SPR sensors and thus holds the potential to detect two biomarkers simultaneously; moreover, the degree of multiplexing could be further increased through the implementation of more sensor elements. Thus, the sensor could enable multiplexing of measurements for the simultaneous detection of various biomarkers. The multiplexing and easy read-out capability with a conventional smartphone could qualify the sensor system for home testing applications, e.g., monitoring of chronic diseases, as well as for differential diagnostics in low resource settings. Current state-of-the-art microfluidic-chip fabrication techniques [26] make the en masse production of the sensors a convenient and cost-effective possibility. Future work on the sensor system will thus include multiplexing as well as the design of a universal sensor housing that can be applied for different types of smartphones, i.e., different dimensions and flashlight LED/camera electronics, with the added advantage of reducing the cost of fabrication.
